# Comparison of Frozen Section and Final Pathology Results in Borderline Ovarian Tumors: A Retrospective Cohort Study

**DOI:** 10.3390/diagnostics16142185

**Published:** 2026-07-14

**Authors:** Isik Sozen, Zeliha Fusun Baba, Ilayda Aksoy, Gokce Nur Esen Topal, Gozde Sahin, Ilkbal Temel Yuksel

**Affiliations:** 1 Department of Gynecologic Oncology, Başakşehir Çam and Sakura City Hospital, 34480 Istanbul, Turkey; gokcenuresen@gmail.com (G.N.E.T.); sahin.gozde1983@gmail.com (G.S.); drilkbaltemel@gmail.com (I.T.Y.); 2 Department of Pathology, Başakşehir Çam and Sakura City Hospital, 34480 Istanbul, Turkey; fusunbaba@yahoo.com; 3 Department of Obstetrics and Gynecology, Başakşehir Çam and Sakura City Hospital, 34480 Istanbul, Turkey; drilaydaaksoy@gmail.com

**Keywords:** borderline ovarian tumor, carcinoma, frozen section, risk factors, ovarian neoplasms

## Abstract

**Background/Objectives:** Borderline ovarian tumor (BOT) is an ovarian neoplasm of low malignant potential that lacks stromal invasion. The aim of this study was to compare intraoperative frozen section analysis (IFSA) findings with final pathology results in patients diagnosed with BOT on frozen sections and to identify risk factors associated with cancer. **Methods:** This study included data from patients who underwent surgery for an ovarian mass between 2020 and 2024 and were diagnosed with BOT on IFSA. Demographic, obstetric, and clinical characteristics, as well as frozen section and final pathology findings, were recorded. The CAR, NLR, and PNI scores were calculated. Patients were grouped as premenopausal or postmenopausal and compared. Based on the final pathology report, patients were also classified as having BOT or cancer and compared. **Results:** A total of 92 patients were included in the study, and 53 (57.6%) were postmenopausal. The prevalence of cancer was significantly higher in the postmenopausal group (*p* = 0.022). Final pathology revealed cancer in 11 patients (11.9%). Age group > 45 years (OR = 12.50) and serous subtype on IFSA (OR = 10.77) were significant risk factors for cancer detection. Cutoff values for distinguishing carcinoma from BOT were identified for CAR (≥1.75), NLR (≥2.64), and PNI (≤48.64). **Conclusions:** In this study, the rate of invasive carcinoma on final pathology among patients diagnosed with BOT on IFSA was 11.9%. Age group > 45 years and serous subtype on IFSA were independent risk factors. The cutoff values for CAR, NLR, and PNI may support risk stratification for carcinoma.

## 1. Introduction

Ovarian masses are commonly encountered in gynecologic practice. Because many of these masses are asymptomatic, their true prevalence in the general population is unclear, and they are often detected incidentally during imaging examinations [1]. The prevalence of benign ovarian cysts varies by age, menstrual status, and ethnicity. The wide variation in reported rates is attributable to differences in the populations studied, the imaging modalities used, and variations in how “simple cyst” versus “complex mass” is defined [2].

Intraoperative frozen section analysis (IFSA) of ovarian masses is a critical tool that provides a rapid preliminary histologic diagnosis during surgery and helps guide the extent of the operation (e.g., fertility-sparing surgery and the need for staging). IFSA results are typically reported as benign, borderline, or malignant [3]. In borderline ovarian tumor (BOT), however, limited sampling may lead to discrepancies between the IFSA diagnosis and the final paraffin-embedded sections [4].

In series of ovarian masses evaluated by IFSA, a diagnosis of BOT is reported less frequently than benign lesions. The proportion of ovarian masses classified as borderline on IFSA is generally around 6–11%. Variation in this rate is largely related to case selection, which depends on the center’s patient profile and the indications for requesting IFSA [5,6].

Detection of BOT on IFSA is not only diagnostically important but also pivotal for intraoperative surgical decision-making. A “BOT” result on frozen section is highly consequential for both the patient and the surgeon, as it enables the surgical extent to be determined during the same procedure and aims to avoid a second operation due to incomplete staging. In patients who desire fertility preservation and are presumed to have early-stage disease, fertility-sparing approaches—such as cystectomy or unilateral salpingo-oophorectomy with preservation of the uterus and contralateral ovary—may be performed [7]. In patients without fertility goals or with a high suspicion of malignancy, total hysterectomy with bilateral salpingo-oophorectomy and comprehensive staging (omentectomy and peritoneal biopsies) is preferred. In mucinous tumors, appendectomy may also be considered [8].

On final pathology, some BOT cases are confirmed as borderline tumors, some are reclassified as benign lesions, and some are “upgraded” to invasive carcinoma [4]. The vast majority of masses reported as BOT on IFSA are concordant with BOT on final pathology. However, when the IFSA report states “at least borderline,” the likelihood of carcinoma on final pathology increases markedly, whereas this rate is lower in cases reported as “straightforward borderline” [3].

Several factors may influence the likelihood of cancer on final pathology in patients diagnosed with BOT on IFSA. The risk of carcinoma is higher in serous tumors and may affect intraoperative decisions regarding the extent of surgery. Mucinous tumors may pose a separate risk because of histologic heterogeneity [3]. In large and complex masses, sampling limitations during IFSA may result in missing invasive foci, increasing the chance of carcinoma on final diagnosis. A multilocular architecture may likewise contribute to missed areas during intraoperative assessment, leading to overlooked malignant foci on frozen section [9]. In addition, freezing artifacts during IFSA can complicate the assessment of stromal invasion [10]. Therefore, the frequency of “upgrade” to cancer in BOT varies with case selection, and surgical planning should be approached cautiously, particularly when IFSA findings are equivocal.

The aim of this study was to compare frozen section findings with final pathology results in patients who underwent surgery for an ovarian mass and were diagnosed with BOT on IFSA, and to identify risk factors associated with cancer.

## 2. Materials and Methods

### 2.1. Patient Selection

The study was conducted using data from patients who underwent surgery for an ovarian mass at the Gynecologic Oncology Surgery Clinic of Başakşehir Çam and Sakura City Hospital between May 2020 and December 2024 and were diagnosed with BOT on IFSA. The study design is a retrospective cohort study.

Patients were included if they were ≥18 years old; underwent surgery for an ovarian mass between 2020 and 2024; had available preoperative blood tests; had preoperative imaging with contrast or non-contrast computed tomography (CT), magnetic resonance imaging (MRI), or ultrasonography (US); underwent IFSA; received a preliminary diagnosis of BOT on IFSA; had a final postoperative pathology evaluation; and had complete data. Patients were excluded if they were <18 years old; lacked preoperative laboratory tests or imaging; did not undergo IFSA; received a non-BOT diagnosis on IFSA; or had missing data.

### 2.2. Data Collection

Patients’ age, obstetric history, presence of comorbidities, preoperative laboratory results, and preoperative imaging findings—including the size of the ovarian mass and laterality—were recorded. Data on the surgical approach (minor vs. major), including fertility-sparing surgery and comprehensive staging surgery, as well as IFSA findings, BOT subtype, and final pathology results were also collected. Obstetric variables included gravida, parity, and history of abortion.

In preoperative laboratory testing, the following values were recorded: white blood cell count, hemoglobin, platelet count, albumin, fibrinogen, C-reactive protein (CRP), cancer antigen (CA) 125, CA 19-9, carcinoembryonic antigen (CEA), and alpha-fetoprotein (AFP). From preoperative imaging, the laterality of the ovarian mass and its size were documented. IFSA results, including the BOT diagnosis and subtype, were also recorded. Using laboratory data, CAR (CRP-to-albumin ratio), NLR (neutrophil-to-lymphocyte ratio), and PNI (prognostic nutritional index) were calculated and recorded.

### 2.3. Study Design

During the study period, patients who underwent surgery for an ovarian mass, received IFSA, and had a preliminary diagnosis of BOT were evaluated. Patients who did not meet the inclusion criteria were excluded. Data from patients diagnosed with BOT on IFSA were recorded. Patients were categorized according to the cohort median age of 45 years as ≤45 years and >45 years. This median-based classification was used only for cohort-specific exploratory comparisons and should not be interpreted as a clinically or biologically validated age threshold in gynecologic oncology. BOT subtypes were classified as serous, mucinous, or other. Surgical procedures were grouped as minor or major.

Patients were grouped according to menopausal status as premenopausal and postmenopausal and were compared. Based on the final pathology report, patients were also classified as the BOT group or the cancer group and compared. Risk factors associated with cancer detection among patients with a BOT diagnosis were identified. Because age and menopausal status were closely related, menopausal status was not included in the final multivariable logistic regression model to reduce collinearity. Age was retained in the model as the clinically and statistically more appropriate variable. Therefore, menopausal status was evaluated only in descriptive and univariate comparisons, whereas independent risk factors were determined using the final multivariable model. To discriminate BOT from carcinoma, cutoff values for CAR, NLR, and PNI were determined using ROC curve analysis.

### 2.4. Intraoperative Frozen Section Analysis (IFSA)

IFSA is a rapid histologic assessment performed within minutes using a frozen section technique on the excised specimen during surgery. In this study, IFSA was performed in all patients according to the institutional routine workflow. After macroscopic evaluation of the surgically excised ovarian mass, tissue samples were obtained from the areas considered most representative of the tumor, including solid, papillary, nodular, necrotic, or invasion-suspicious regions. In large, multilocular, or heterogeneous masses, the number of sampled sections was increased to reduce the risk of missing invasive foci. The tissue samples were frozen using the cryostat method, thin sections were prepared, and rapid hematoxylin-eosin staining was performed before evaluation by an experienced pathologist. In ovarian tumors, IFSA helps guide the extent of surgery by enabling intraoperative distinction between benign, borderline, and malignant lesions [11].

When prominent epithelial proliferation and nuclear atypia were present but destructive stromal invasion could not be demonstrated, the lesion was reported in favor of a borderline tumor. Although IFSA is useful in BOT, complete concordance with final pathology is not expected, and some cases may be upgraded to invasive carcinoma on permanent sections. Therefore, IFSA findings should be interpreted cautiously, and the definitive diagnosis was confirmed in all cases by detailed histopathological examination of permanent paraffin-embedded [3].

### 2.5. Surgical Technique and Grouping

Surgical management of ovarian masses varied according to the patient’s clinical status. Depending on the surgeon’s preference, procedures included ovarian cystectomy, salpingo-oophorectomy, and hysterectomy with bilateral salpingo-oophorectomy, with staging procedures performed when indicated in conjunction with these techniques. In this study, all patients underwent surgery. Patients were grouped as major surgery (hysterectomy with bilateral salpingo-oophorectomy) or minor surgery (ovarian cystectomy, salpingo-oophorectomy).

### 2.6. Histopathological Examination

On IFSA, the tumor is assessed intraoperatively using a limited number of rapid sections. When prominent epithelial proliferation and nuclear atypia are present but destructive stromal invasion cannot be demonstrated, the lesion is interpreted as borderline, and in equivocal cases, intermediate terms such as “at least borderline” may be used [3]. If destructive stromal invasion is identified, a diagnosis of invasive carcinoma is made. In the absence of invasion, the BOT diagnosis is confirmed according to the histologic subtype; however, due to the sampling limitations of IFSA, definitive classification is based on the permanent paraffin-embedded sections [7].

In this study, frozen section and final pathology specimens were evaluated by experienced pathologists. Based on the final pathology results, patients with a diagnosis of borderline tumor or “at least borderline” were classified as the BOT group, whereas patients with stromal invasion were classified as the cancer group.

### 2.7. CRP-to-Albumin Ratio (CAR)

CAR is calculated by dividing the serum CRP level (mg/L) by the serum albumin level (g/L). CAR strengthens inflammation-based prognostic stratification in patients with cancer, and a higher CAR predicts worse overall survival [12]. Discriminative performance was assessed using receiver operating characteristic (ROC) curve analysis; the area under the curve (AUC) was calculated, and sensitivity and specificity were reported.

### 2.8. Neutrophil-to-Lymphocyte Ratio (NLR)

NLR was calculated by dividing the absolute neutrophil count by the absolute lymphocyte count obtained from a complete blood count. NLR provides risk stratification based on the degree of systemic inflammation in patients with cancer, and a higher NLR is considered a marker of poorer survival [13]. Discriminative performance was evaluated using ROC curve analysis; the area under the curve (AUC) was calculated, and sensitivity and specificity were reported.

### 2.9. Prognostic Nutritional Index (PNI)

PNI was calculated using the following formula: 10 × serum albumin (g/dL) + 0.005 × total lymphocyte count (/µL). PNI provides risk stratification based on immuno-nutritional status in patients with cancer. A lower PNI predicts worse overall survival across many tumor types [14]. In this study, the discriminative performance of CAR, NLR, and PNI was evaluated using ROC curve analysis. For each score, the AUC was calculated to assess discrimination of patients with cancer, and sensitivity and specificity were reported.

### 2.10. Ethics Approval and Consent to Participate:

All procedures performed in studies involving human participants were conducted in accordance with the ethical standards of the institutional and/or national research committee and with the 1964 Declaration of Helsinki and its later amendments. The study was approved by the Başakşehir Çam and Sakura City Hospital Ethics Committee (approval date: 12 February 2025; approval number: 46).

### 2.11. Statistical Analysis

Data distribution was assessed using the Shapiro–Wilk and Kolmogorov–Smirnov tests, and variables were classified as parametric or nonparametric. Parametric continuous variables were summarized as mean (x) and standard deviation (SD), whereas nonparametric continuous variables were summarized as median and interquartile range (Q1–Q3). Categorical variables were expressed as frequency (*n*) and percentage (%). An independent-samples *t* test was used to compare means between two independent groups, and the Mann–Whitney U test was used to compare medians for nonparametric variables. Associations between categorical variables were evaluated using the chi-square test or Fisher’s exact test, as appropriate. Risk factors for the presence of cancer among patients with BOT were explored using backward elimination and enter methods. Logistic regression analysis was performed using statistically significant variables. The relationship between radiologic and pathologic tumor size was assessed using Pearson correlation analysis. Receiver operating characteristic (ROC) curve analysis was used to evaluate the ability of CAR, NLR, and PNI to detect cancer. In ROC analysis, statistical significance and diagnostic performance were determined based on sensitivity, specificity, positive likelihood ratio (LR+), and negative likelihood ratio (LR−).

The analyses in the tables were designed as descriptive and exploratory group comparisons rather than confirmatory hypothesis tests. Therefore, multiple comparison correction was not applied; findings were interpreted by considering *p*-values and effect sizes together. Furthermore, due to the limited number of patients in the carcinoma group, it was considered that conservative corrections might increase the risk of type II errors. Therefore, univariate results were considered exploratory, and the main interpretations were made in line with multivariate analysis and clinical significance.

Before multivariable logistic regression, univariate analyses were performed to evaluate the association between each candidate variable and carcinoma detection on final pathology. Variables with statistical significance in univariate analysis and/or clinical relevance were considered for inclusion in the multivariable model. A *p* value < 0.05 was considered statistically significant. All analyses were performed using SPSS version 25.0 (Armonk, New York, NY, USA).

## 3. Results

A total of 92 patients were included in the study. The mean age was 46.65 ± 8.99 years, with a median age of 45 years. The mean BMI was 30.73 ± 4.65 kg/m^2^. Of the patients, 39 (42.4%) were premenopausal and 53 (57.6%) were postmenopausal. Patients were grouped as premenopausal and postmenopausal and compared.

The mean age was 52.62 ± 6.80 years in the postmenopausal group and 38.54 ± 5.31 years in the premenopausal group. The mean BMI was 32.28 ± 4.92 kg/m^2^ in the postmenopausal group and 28.62 ± 3.39 kg/m^2^ in the premenopausal group. Both age and BMI were significantly higher in the postmenopausal group than in the premenopausal group (*p* < 0.001 for both).

**Figure 1:**
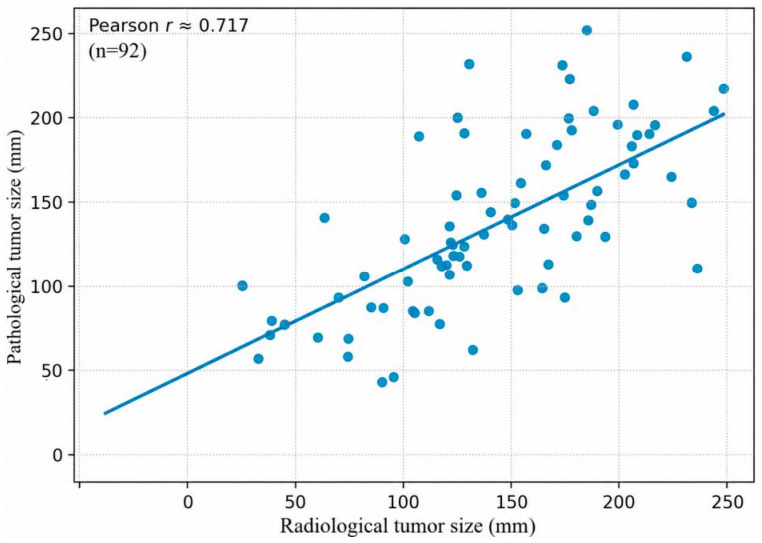
Radiologic–pathologic correlation of ovarian tumor size.

When all patients were evaluated, the mean radiological tumor size was found to be 128.53 ± 60.56 mm, and the mean pathological tumor size was 124.04 ± 59.30 mm. A strong positive correlation was observed between radiological and pathological tumor sizes (r = 0.717, *p* < 0.001) (Figure 1). This finding indicates that the tumor size determined by preoperative imaging is generally consistent with the final pathological measurement. When patients were grouped by menopausal status, there was no significant difference between the groups in radiologic or pathologic tumor size (Table 1).

In the postmenopausal group, 44 patients (83.0%) were in the >45 years age group, compared with 2 patients (5.1%) in the premenopausal group. A history of comorbid disease was present in 27 patients (50.9%) in the postmenopausal group and in 9 patients (23.1%) in the premenopausal group. Major surgery was performed in 51 patients (96.2%) in the postmenopausal group and in 5 patients (12.8%) in the premenopausal group. The frequencies of the >45 years age group, comorbid disease, and major surgery were significantly higher in the postmenopausal group than in the premenopausal group (*p* < 0.001, *p* = 0.007, and *p* < 0.001, respectively) (Table 1).

When patients were grouped by menopausal status, there were no statistically significant differences between the groups in gravida, parity, history of abortion, laterality of the ovarian mass, or the distribution of BOT subtypes. Cancer was identified on final pathology in 10 patients (18.9%) in the postmenopausal group and in one patient (2.6%) in the premenopausal group. The frequency of cancer detection was significantly higher in the postmenopausal group than in the premenopausal group (*p* = 0.022) (Table 1).

Cancer was diagnosed on final pathology in 11 patients (11.9%). Accordingly, 11 patients (11.9%) were classified as the cancer group and 81 patients (88.1%) as the BOT group. The mean age was 53.14 ± 4.43 years in the cancer group and 45.77 ± 9.31 years in the BOT group. Mean age was significantly higher in the cancer group than in the BOT group (*p* < 0.001). There were no statistically significant differences between the groups in BMI or in mean radiologic and pathologic tumor size (Table 2).

In the cancer group, 10 patients (90.9%) were in the >45 years age group, compared with 36 patients (44.4%) in the BOT group. Postmenopausal status was present in 10 patients (90.9%) in the cancer group and 43 patients (53.1%) in the BOT group. Final pathology results were further evaluated according to histopathological subtypes. In the BOT group, final pathology revealed serous BOT in 39 patients (48.1%), mucinous BOT in 39 patients (48.1%), endometrioid BOT in 2 patients (2.6%), and clear cell BOT in 1 patient (1.2%). In the carcinoma group, final pathology revealed serous carcinoma in 10 patients (90.9%) and mucinous carcinoma in 1 patient (9.1%). Among the ten patients with serous carcinoma in the carcinoma group, two had been classified as low-grade serous carcinoma (LGSC) in the BOT group based on IFSA. Therefore, these cases were additionally recorded as showing an upgrade to low-grade serous carcinoma in the final pathology. All patients in the cancer group underwent major surgery (Table 2).

There were no significant differences between the groups with respect to the laboratory parameters, except for CA 125 (*p* > 0.05). The median CA 125 level was 39.5 (21.5–85.2) in the cancer group and 32.7 (17.5–70.6) in the BOT group. CA 125 was significantly higher in the cancer group than in the BOT group (*p* = 0.021) (Table 3).

The variables evaluated before multivariable modeling are presented. In univariate analyses, older age, postmenopausal status, major surgery, CA-125 level, and histopathological subtype-related variables showed associations with carcinoma detection. However, because of collinearity with age, menopausal status was not entered into the final multivariable model.

Risk factors associated with cancer detection were evaluated using multivariable analysis. Age group > 45 years (OR = 12.50; 95% CI: 1.53–102.27) and having the serous subtype on IFSA (OR = 10.77; 95% CI: 1.32–88.06) were statistically significant risk factors for cancer detection (Table 4).

**Figure 2:**
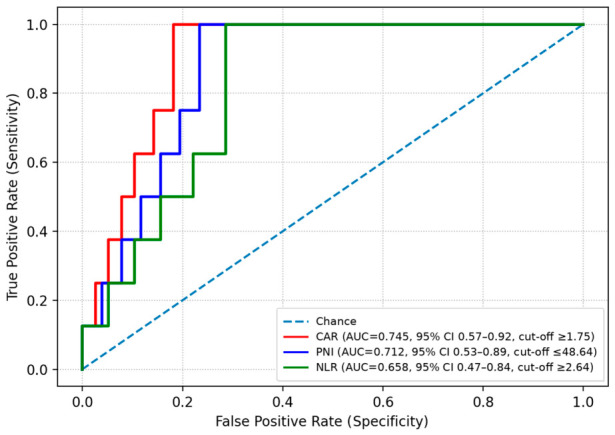
ROC curve analysis of CAR, NLR, and PNI for detecting carcinoma among patients diagnosed with borderline ovarian tumors on IFSA. CAR showed an AUC of 0.745 (95% CI: 0.57–0.92), NLR showed an AUC of 0.658 (95% CI: 0.47–0.84), and PNI showed an AUC of 0.712 (95% CI: 0.53–0.89).

ROC curve analysis was performed to assess the ability of CAR, NLR, and PNI to distinguish carcinoma within the BOT cohort. For identifying carcinoma in patients with BOT, CAR ≥ 1.75 yielded a sensitivity of 75.0%, a specificity of 81.8%, and an AUC of 0.745. For NLR ≥ 2.64, sensitivity was 62.5%, specificity was 71.4%, and the AUC was 0.658. For PNI ≤ 48.64, sensitivity was 75.0%, specificity was 76.6%, and the AUC was 0.712 (Table 5) (Figure 2).

## 4. Discussion

In the present study, invasive carcinoma was detected on final pathology in 11.9% of patients who had been diagnosed with BOT on IFSA. This finding is generally consistent with the approximately 8–12% upgrade rate reported in the literature and systematic reviews [3]. De Decker et al. reported an invasive carcinoma rate of 7.7% on permanent paraffin sections after an IFSA diagnosis of BOT [15]. Previous studies have also suggested that malignant upgrade may be more frequent in postmenopausal patients [16]. Therefore, considering the relatively high proportion of postmenopausal patients in our cohort, the observed 11.9% rate appears to be compatible with previously reported data, although it should be interpreted cautiously given the exploratory nature of the study and the small number of carcinoma cases.

The frequency of BOT detection on IFSA may vary by menopausal status. In the study by Shen et al., BOT was reported more often in the premenopausal age group, with approximately 80% of cases diagnosed before menopause [17]. In contrast, Pecorino et al. reported a higher proportion of postmenopausal cases, with the rate increasing to 33–36% in menopause [18]. In our study, 57.6% of patients were postmenopausal.

Histologic subtypes of BOTs most commonly include serous, mucinous, seromucinous, and other variants. In the study by Shen et al., the distribution of serous/mucinous/other subtypes was similar according to menopausal status. In contemporary series, the proportion of postmenopausal cases has reached a notable level, with approximately 20–30% reported [17]. Similarly, in the present study, there was no significant difference in the distribution of BOT subtypes between the premenopausal and postmenopausal groups (*p* = 0.499).

Differences in the number of pregnancies, deliveries, and abortions according to menopausal status have been evaluated in BOT. In the study by Baykus et al., the number of pregnancies, deliveries, and history of abortion were reported to be similar between menopausal groups [19]. Likewise, in the present study, there were no differences in the distribution of obstetric variables between the groups according to menopausal status (*p* > 0.05).

The choice of surgical approach in BOT may differ according to menopausal status. More extensive procedures, such as hysterectomy with bilateral oophorectomy, are performed more frequently in postmenopausal patients. In premenopausal patients, fertility-sparing surgery is more commonly preferred, with cystectomy or unilateral salpingo-oophorectomy being the predominant approaches [16]. In our study, the frequency of major surgery was significantly higher in the postmenopausal group (*p* < 0.001).

In patients diagnosed with BOT on IFSA who are found to have invasive carcinoma on final pathology, the mean age is typically higher, and the proportion of postmenopausal patients tends to increase [16]. In the present study, both the mean age and the frequency of patients in the >45 years age group was significantly higher in the carcinoma group (*p* < 0.001 and *p* = 0.007, respectively). In the study by Sagır et al., the prevalence of the >45 years age group increased markedly in the postmenopausal group and independently increased the risk of frozen–paraffin diagnostic discordance (OR = 5.06; 95% CI: 3.47–7.36) [20]. Similarly, in our study, Age group > 45 years was a significant risk factor for carcinoma among patients with BOT (OR = 12.50; 95% CI: 1.53–102.27). These findings suggest that higher age within this cohort may be associated with increased clinical suspicion; however, the median-based age category used in this study was cohort-specific and should not be interpreted as a validated clinical cutoff for older age.

The proportion of postmenopausal patients is higher in the group diagnosed with invasive carcinoma on final pathology [16]. In the present study, the frequency of postmenopausal status was significantly higher in the carcinoma group in univariate comparison (*p* = 0.022). However, menopausal status was not included in the final multivariable model because of its collinearity with age. Therefore, this finding should be interpreted as an exploratory group difference rather than an independent risk factor. In the final multivariable analysis, only age > 45 years and serous subtype on IFSA remained statistically significant risk factors for carcinoma detection.

Histopathologic subtype is an important factor associated with the detection of carcinoma on final pathology in BOT. In the study by De Decker et al., the carcinoma rate in serous tumors was 3.1% when the IFSA diagnosis was BOT. In the same study, among serous tumors with an IFSA report of “at least borderline,” the carcinoma rate was 35.7% [15]. In the present study, the frequency of the serous subtype was significantly higher in the carcinoma group (*p* = 0.022).

In cases diagnosed with BOT on IFSA that are subsequently found to have invasive carcinoma on final pathology, comprehensive staging surgery is performed more frequently. In the study by Raimondo et al., a more aggressive surgical approach was more common in postmenopausal patients, and bilateral salpingo-oophorectomy was performed at higher rates [21]. In the present study, the frequency of comprehensive and major surgery was significantly higher in the carcinoma group (*p* = 0.001).

Tumor size is a factor associated with the detection of invasive carcinoma on final pathology in patients with BOT. Large masses can lead to representativeness issues on IFSA, and invasive foci may be missed if they fall outside the sampled sections. In the study by Huang et al., underdiagnosis was more common in tumors larger than 10 cm [22]. Increased tumor volume and marked heterogeneity have also been identified as independent factors that increase frozen–paraffin diagnostic discordance [23]. However, in the present study, there was no significant difference in tumor size between groups according to the presence of carcinoma (*p* = 0.667).

Gravidity, parity, and a history of abortion were similar between patients in whom invasive carcinoma was identified and those in whom BOT was confirmed on final pathology. In the study by Park et al., parity was not shown to be associated with an upgrade of the IFSA diagnosis on final pathology [9]. The relationship between reproductive factors and BOT has been reported heterogeneously; nulliparity has been associated with recurrence risk in some cohorts [24]. Similarly, in the present study, there were no significant differences between the groups according to carcinoma status (*p* > 0.05).

The ability of routine blood tests to predict final pathology results and to discriminate between outcomes is limited. In the study by Akalin et al., patients with invasive carcinoma were compared with those whose borderline diagnosis was confirmed, and preoperative serum biochemical parameters and CA 19-9 levels were similar between the groups [25]. In the present study, laboratory parameters did not have a decisive role in explaining frozen–paraffin discordance between groups. The diagnostic performance of serum tumor markers is limited, and they should not be used as stand-alone tools in clinical decision-making.

In contrast, preoperative CA 125 may be an exception for identifying carcinoma. In the study by Zhang et al., preoperative CA 125 levels were higher in the group with invasive carcinoma. In mucinous BOT, a relatively elevated CA 125 level has been reported among variables predicting malignancy after an IFSA diagnosis [26]. Similarly, in the present study, CA 125 was significantly higher in the carcinoma group (*p* = 0.021). Nevertheless, CA 125 is nonspecific and may also be elevated in benign gynecologic conditions. Thus, differences in CA 125 can support clinical risk assessment but are not sufficient as a stand-alone determinant for surgical decision-making.

In the exploratory univariate analyses, CA-125 levels were higher in the carcinoma group than in the BOT group. However, this finding should be interpreted cautiously because multiple univariate comparisons were performed without formal adjustment, and the carcinoma subgroup was small. CA-125 is also nonspecific and may be elevated in benign gynecologic conditions. Therefore, the observed difference in CA-125 should be regarded as a hypothesis-generating finding that may support, but cannot independently determine, clinical risk assessment. Validation in larger prospective cohorts is required before CA-125 can be considered a reliable marker for carcinoma detection in this setting.

Histologic subtype has a substantial impact on the risk of upgrade to invasive carcinoma on final pathology in BOT diagnosed on IFSA. In the study by Shah et al., mucinous tumors had a higher risk of upgrade than serous tumors (OR = 7.1; 95% CI: 2.1–23.7) [4]. In the study by Chen et al., clear cell/endometrioid subtypes had a markedly higher likelihood of upgrade to invasive carcinoma compared with serous tumors (OR = 48.1; 95% CI: 8.8–261.8) [27]. In the present study, being in the serous subtype on IFSA was a significant risk factor for carcinoma among patients with BOT (OR = 10.77; 95% CI: 1.32–88.06).

Systemic inflammation-based scores may provide additional information for predicting invasive cancer on final pathology. For NLR, an ROC-derived cutoff of 2.42 has been reported for distinguishing BOT from epithelial ovarian cancer, and NLR > 2.42 was associated with an increased likelihood of malignancy (OR = 2.36; 95% CI: 1.19–4.68) [28]. In the study by Song et al., an NLR cutoff of 2.92 was identified for differentiating BOT from ovarian cancer, with an AUC of 0.728 [29].

Low PNI has been associated with malignancy, and a PNI cutoff of 46.90 has been reported [29]. In the study by Barik et al., CAR was associated with disease severity in ovarian cancer, and an ROC-derived cutoff of 1.08 indicated advanced-stage disease [30]. No validated CAR cutoff has been established specifically for BOT, and most available evidence comes from invasive cancer cohorts. In the present study, cutoff values for these scores were determined to distinguish patients diagnosed with carcinoma on final pathology among those with an IFSA diagnosis of BOT. The identified cutoffs were NLR ≥ 2.64, PNI ≤ 48.64, and CAR ≥ 1.75. These scores should be considered as potentially useful adjuncts for post-IFSA risk stratification.

CAR, NLR, and PNI are not tumor-specific markers; rather, they are general biomarkers influenced by systemic inflammation, immune response, and nutritional status. Therefore, they should not be used as stand-alone determinants to guide surgical decision-making. Instead, they should be considered as adjunctive risk stratification tools together with age, menopausal status, imaging findings, IFSA results, and histopathological subtypes. The limited number of patients in the cancer group reduces the statistical power of the ROC curve analyses performed for CAR, NLR, and PNI. Therefore, the obtained cut-off, AUC, sensitivity, and specificity values should be interpreted with caution.

A key strength of this study is that it directly links the detection of cancer on final pathology in patients diagnosed with BOT on IFSA to the clinical decision-making process. Comparing frozen section and permanent paraffin results provides an objective assessment of diagnostic upgrade. Evaluating readily available variables—such as age, menopausal status, histologic subtype, and CA-125—together may offer a practical contribution to intraoperative risk stratification.

The main limitations of this study are its retrospective and single-center design, the small number of patients in the carcinoma group (*n* = 11), and the resulting limited statistical power. In particular, the low number of carcinoma cases may have made it difficult for the multivariable logistic regression analysis to fully meet standard statistical assumptions and may have limited the reliability of the estimates obtained. In addition, potential collinearity among variables such as age, menopausal status, and type of surgery may affect the interpretation of the multivariable model. The absence of a standardized protocol for IFSA tissue sampling and the lack of detailed data on the expertise level of the pathologists who evaluated IFSA also limit the assessment of diagnostic concordance. Finally, the ROC-derived cut-off values determined for CAR, NLR, and PNI were not validated in an independent external cohort. Therefore, these findings should be regarded not as direct clinical decision-making tools, but as preliminary results that require confirmation in prospective multicenter studies.

Because the primary outcome was the observed rate of carcinoma detection on final pathology among patients diagnosed with BOT on IFSA, a conventional post hoc power analysis was not considered appropriate in the absence of a pre-specified comparator, effect size, or hypothesis-testing framework. Instead, the precision of the primary outcome estimate was reported. In this cohort, carcinoma was detected in 11 of 92 patients, corresponding to an observed rate of 11.9% with a 95% confidence interval of approximately 6.8–20.2%. This relatively wide confidence interval reflects the limited precision resulting from the small number of carcinoma events and supports interpreting the findings as exploratory and hypothesis-generating.

In the future, larger study groups must be examined to validate the findings reported here. Based on a two-sided alpha level of 0.05 and 80% power, future validation studies should include at least 390 participants, approximately 195 per comparison group, and preferably around 430 participants to account for incomplete data.

Future studies should be designed as prospective, multicenter investigations. A standardized IFSA sampling protocol should be implemented, and the surgical decision-making algorithm should be clearly defined. ROC-derived cutoff values for NLR, PNI, CAR, and CA-125 should be validated in independent cohorts.

## 5. Conclusions

In this study, frozen section and final pathology results were compared in patients diagnosed with BOT on IFSA. The rate of invasive carcinoma on final pathology was 11.9%. Age group > 45 years and serous subtype on IFSA were more frequent in the carcinoma group. In the final multivariable model, age group > 45 years (OR = 12.50) and serous subtype on IFSA (OR = 10.77) were independent risk factors for cancer detection on final pathology. ROC analysis demonstrated moderate discriminative performance for the thresholds of CAR ≥ 1.75, NLR ≥ 2.64, and PNI ≤ 48.64, which may support post-IFSA risk stratification.

## Figures and Tables

**Table 1:** Grouping and comparison of patients according to menopausal status.

	All Patients (*n* = 92)	Premenopausal Group (*n* = 39)	Postmenopausal Group (*n* = 53)	*p* Value	Effect Size (Cohen’s d)
	x ± SD	x ± SD	x ± SD		
**Age (years)**	46.65 ± 8.99	38.54 ± 5.31	52.62 ± 6.80	<0.001 *	2.27
**BMI (kg/m^2^)**	30.73 ± 4.65	28.62 ± 3.39	32.28 ± 4.92	<0.001 *	0.84
**Tumor size**					
Radiological (mm)	128.53 ± 60.56	131.26 ± 61.45	126.52 ± 60.31	0.713 *	0.08
Pathological (mm)	124.04 ± 59.30	128.88 ± 60.17	120.48 ± 58.72	0.506 *	0.14
	***n*** **(%)**	***n*** **(%)**	***n*** **(%)**		**Effect Size** **(Cramer’s V)**
**Age group, years**					
≤45	46 (50)	37 (94.9)	9 (17.0)	<0.001 **	0.770
>45	46 (50)	2 (5.1)	44 (83.0)		
**Pregnancy**					
Yes	77 (83.7)	31 (79.5)	46 (86.8)	0.277 **	0.113
No	15 (16.3)	8 (20.5)	7 (13.2)		
**Birth**					
Yes	74 (80.4)	29 (74.4)	45 (84.9)	0.249 **	0.128
No	18 (19.6)	10 (25.6)	8 (15.1)		
**History of abortion**					
Yes	17 (18.5)	6 (15.4)	11 (20.8)	0.512 **	0.068
No	75 (81.5)	33 (84.6)	42 (79.2)		
**Comorbid disease**					
Yes	36 (39.1)	9 (23.1)	27 (50.9)	0.007 **	0.304
No	56 (60.9)	30 (76.9)	26 (49.1)		
**Location of ovarian mass**					
Right	45 (48.9)	20 (51.3)	25 (47.2)	0.637 ***	0.104
Left	39 (42.4)	17 (43.6)	22 (41.5)		
Bilateral	8 (8.7)	2 (5.1)	6 (11.3)		
**Surgery performed**					
Major surgery	56 (60.9)	5 (12.8)	51 (96.2)	<0.001 **	0.845
Minor surgery	36 (39.1)	34 (87.2)	2 (3.8)		
**Subtypes of BOT according to frozen section examination**					
Serous	49 (53.3)	19 (48.7)	30 (56.6)	0.672 ***	0.090
Mucinous	40 (43.5)	19 (48.7)	21 (39.6)		
Other	3 (3.2)	1 (2.6)	2 (3.8)		
**Final pathology report**					
BOT	81 (88.1)	38 (97.4)	43 (81.1)	0.022 ***	0.248
Carcinoma	11 (11.9)	1 (2.6)	10 (18.9)		

BOT: borderline ovarian tumor, kg: kilogram, m^2^: square meter, mm: millimeter, *n*: frequency, SD: standard deviation, x: mean. * Independent *t*-test, ** Chi-square, *** Fisher exact, *p* < 0.05.

**Table 2:** Grouping and comparison of patients according to their pathology report.

	All Patients (*n* = 92)	BOT Group (*n* = 81)	Cancer Group (*n* = 11)	*p* Value	Effect Size (Cohen’s d)
	x ± SD	x ± SD	x ± SD		
**Age (years)**	46.65 ± 8.99	45.77 ± 9.31	53.14 ± 4.43	<0.001	0.834
**BMI (kg/m^2^)**	30.73 ± 4.65	30.81 ± 4.74	30.12 ± 4.23	0.625	0.152
**Tumor size**					
Radiological (mm)	128.53 ± 60.56	129.28 ± 60.97	122.98 ± 59.43	0.747	0.104
Pathological (mm)	124.04 ± 59.30	124.94 ± 60.42	117.45 ± 51.87	0.667	0.129
	***n*** **(%)**	***n*** **(%)**	***n*** **(%)**		**Effect Size** **(Cramer’s V)**
**Age group, years**					
≤45	46 (50)	45 (55.6)	1 (9.1)	0.007	0.302
>45	46 (50)	36 (44.4)	10 (90.9)		
**Menopause**					
Premenopause	39 (42.4)	38 (46.9)	1 (9.1)	0.022	0.248
Postmenopause	53 (57.6)	43 (53.1)	10 (90.9)		
**Pregnancy**					
Yes	77 (83.7)	69 (85.2)	8 (72.7)	0.379	0.109
No	15 (16.3)	12 (14.8)	3 (27.3)		
**Birth**					
Yes	74 (80.4)	67 (82.7)	7 (63.6)	0.216	0.156
No	18 (19.6)	14 (17.3)	4 (36.4)		
**History of abortion**					
Yes	17 (18.5)	13 (16.1)	4 (36.4)	0.116	0.170
No	75 (81.5)	68 (83.9)	7 (63.6)		
**Comorbid disease**					
Yes	36 (39.1)	30 (37.1)	6 (54.5)	0.329	0.116
No	56 (60.9)	51 (62.9)	5 (45.5)		
**Location of ovarian mass**					
Right	45 (48.9)	40 (49.4)	5 (45.5)	0.489	0.125
Left	39 (42.4)	35 (43.2)	4 (36.4)		
Bilateral	8 (8.7)	6 (7.4)	2 (18.2)		
**Surgery performed**					
Major surgery	56 (60.9)	45 (55.6)	11 (100)	0.001	0.285
Minor surgery	36 (39.1)	36 (44.4)	-		
**Subtypes of BOT according to frozen section examination**					
Serous	49 (53.3)	39 (48.1)	10 (90.9)	0.005	0.340
Mucinous	40 (43.5)	39 (48.1)	1 (9.1)		
Other	3 (3.2)	3 (3.8)	-		
**Final histopathology results**					
Serous	49 (53.3)	39 (48.1)	10 (90.9)	0.003	0.392
Mucinous	40 (43.5)	39 (48.1)	1 (9.1)		
Endometroid	2 (2.2)	2 (2.6)	-		
Clear cell	1 (1.1)	1 (1.2)	-		

Note: The univariate comparisons presented in this table are exploratory and hypothesis-generating. No formal multiple-comparison correction was applied; therefore, *p* values should be interpreted cautiously and not as confirmatory evidence. Effect sizes are provided to support clinical interpretation. BOT: borderline ovarian tumor, kg: kilogram, m^2^: square meter, mm: millimeter, *n*: frequency, SD: standard deviation, x: mean.

**Table 3:** Comparison of blood test results of patients by group.

	All Patients (*n* = 92)	BOT Group (*n* = 81)	Cancer Group (*n* = 11)	*p* Value	Effect Size
	x ± SD	x ± SD	x ± SD		
WBC	8.3 ± 2.4	8.4 ± 2.4	7.6 ± 2.2	0.283	0.34
Hbg	12.3 ± 1.8	12.3 ± 1.8	12.4 ± 1.6	0.851	0.06
PLT	294.8 ± 67.5	297.4 ± 68.7	275.9 ± 57.7	0.277	0.32
Albumin	4.2 ± 0.9	4.2 ± 0.9	3.9 ± 0.6	0.165	0.34
Fibrinogen	371.2 ± 97.7	365.2 ± 96.2	415.5 ± 104.7	0.156	0.52
	**Median (Q1–Q3)**	**Median (Q1–Q3)**	**Median (Q1–Q3)**	***p*** ** Value**	
CRP	3.4 (1.0–5.7)	3.1 (1.0–4.5)	4.2 (1.0–6.4)	0.428	0.086
CA 125	33.5 (18.3–71.5)	32.7 (17.5–70.6)	39.5 (21.5–85.2)	0.021	0.250
CEA	1.7 (0.9–2.8)	1.7 (0.9–2.7)	2.1 (1.4–3.8)	0.613	0.055
CA 19.9	15.1 (6.2–31.7)	14.8 (6.1–30.4)	15.4 (6.9–36.2)	0.524	0.069
AFP	2.2 (1.4–3.6)	2.1 (1.4–3.4)	2.2 (2.0–3.6)	0.487	0.075

AFP: alpha-fetoprotein, CA: cancer antigen, CEA: carcinoembryonic antigen, CRP: C-reactive protein, Hgb: hemoglobin, PLT: platelet, SD: standard deviation, Q: quartile, WBC: white blood cell, x: mean.

**Table 4:** Final multivariable logistic regression model for carcinoma detection among patients diagnosed with borderline ovarian tumors on frozen section analysis.

	Odds Ratio	95% Confidence Interval	*p* Value
Age (>45 years)	12.50	1.53–102.27	0.018
BOT subgroup (serous)	10.77	1.32–88.06	0.027

BOT: borderline ovarian tumor, *p* < 0.05.

**Table 5:** Cut-off values of scores for detecting carcinomas within borderline ovarian tumors.

	Sensitivity	Specificity	AUC	95% CI	LR+	LR−	Cut-Off	*p* Value
CAR	75.0	81.8	0.745	0.57–0.92	4.12	0.31	≥1.75	<0.001
NLR	62.5	71.4	0.658	0.47–0.84	2.19	0.53	≥2.64	<0.001
PNI	75.0	76.6	0.712	0.53–0.89	3.21	0.33	≤48.64	<0.001

AUC: area under the curve, CAR: CRP/albumin ratio, LR: likelihood ratio, NLR: neutrophil/lymphocyte ratio, PNI: prognostic nutrition index.

## Data Availability

The data presented in this study are available on request from the corresponding author due to privacy and ethical reasons.

## Abbreviations

The following abbreviations are used in this manuscript:

BOT	Borderline ovarian tumor
IFSA	Intraoperative frozen section analysis
CAR	CRP-to-albumin ratio
NLR	Neutrophil-to-lymphocyte ratio
PNI	prognostic nutritional index
CT	Computed tomography
MRI	Magnetic resonance imaging
US	Ultrasonography
CRP	C-reactive protein
CA	cancer antigen
CEA	carcinoembryonic antigen
AFP	Alpha-fetoprotein
ROC	Receiver operating characteristic
AUC	Area under the curve
BMI	Body mass index

## References

[1] Carvalho J.P., Moretti-Marques R., Filho A.L.D.S. (2020). Adnexal mass: Diagnosis and management. Rev. Bras. Ginecol. Obstet..

[2] Mansour S., Hamed S., Kamal R. (2023). Spectrum of Ovarian Incidentalomas: Diagnosis and Management. Br. J. Radiol..

[3] De Decker K., Jaroch K.H., Edens M.A., Bart J., Kooreman L.F.S., Kruitwagen R.F.P.M., Nijman H.W., Kruse A.J. (2021). Frozen section diagnosis of borderline ovarian tumors with suspicious features of invasive cancer is a devil’s dilemma for the surgeon: A systematic review and meta-analysis. Acta Obstet. Gynecol. Scand..

[4] Shah J.S., Mackelvie M., Gershenson D.M., Ramalingam P., Kott M.M., Brown J., Gauthier P., Nugent E., Ramondetta L.M., Frumovitz M. (2019). Accuracy of Intraoperative Frozen Section Diagnosis of Borderline Ovarian Tumors by Hospital Type. J. Minim. Invasive Gynecol..

[5] Muruthapongsatorn P., Inploy N., Prommas S., Smanchat B., Bhamarapravatana K., Suwannarurk K. (2019). The Evaluation of Intra-Operative Frozen Section Diagnosis Accuracy of Ovarian Tumors; Old Fashioned Not out of Fashion. Asian Pac. J. Cancer Prev..

[6] Pujani M., Raychaudhuri S., Singh K., Agarwal C., Jain M., Chauhan V., Sidam D., Chandoke R.K., Sharma J.C., Sharma P. (2023). A Critical Appraisal of Intraoperative Frozen Section Analysis of Ovarian Tumors: A 3-Year Review of Accuracy and Clinicopathological Correlation at a Tertiary Care Center. J. Microsc. Ultrastruct..

[7] Della Corte L., Mercorio A., Serafino P., Viciglione F., Palumbo M., De Angelis M.C., Borgo M., Buonfantino C., Tesorone M., Bifulco G. (2022). The challenging management of borderline ovarian tumors (BOTs) in women of childbearing age. Front. Surg..

[8] Maramai M., Barra F., Menada M.V., Stigliani S., Moioli M., Costantini S., Ferrero S. (2020). Borderline ovarian tumours: Management in the era of fertility-sparing surgery. Ecancermedicalscience.

[9] Park J.Y., Lee S.H., Kim K.R., Kim Y.T., Nam J.H. (2019). Accuracy of frozen section diagnosis and factors associated with final pathological diagnosis upgrade of mucinous ovarian tumors. J. Gynecol. Oncol..

[10] Gupta A., Jain N. (2021). Evaluation of a step-by-step approach to frozen section diagnosis in ovarian masses. Asian J. Med. Sci..

[11] Colombo N., Sessa C., Bois A.D., Ledermann J., McCluggage W.G., McNeish I., Morice P., Pignata S., Ray-Coquard I., Vergote I. (2019). ESMO-ESGO consensus conference recommendations on ovarian cancer: Pathology and molecular biology, early and advanced stages, borderline tumours and recurrent disease. Int. J. Gynecol. Cancer.

[12] Liao C.K., Yu Y.L., Lin Y.C., Hsu Y.J., Chern Y.J., Chiang J.M., You J.F. (2021). Prognostic value of the C-reactive protein to albumin ratio in colorectal cancer: An updated systematic review and meta-analysis. World J. Surg. Oncol..

[13] Guo Y., Xiang D., Wan J., Yang L., Zheng C. (2022). Focus on the Dynamics of Neutrophil-to-Lymphocyte Ratio in Cancer Patients Treated with Immune Checkpoint Inhibitors: A Meta-Analysis and Systematic Review. Cancers.

[14] Chen J., Jin L., Luo R., Zhang X., Chen Y., Han Z., Liu T. (2025). Predictive value of preoperative systemic immune-inflammation index and prognostic nutrition index in patients with epithelial ovarian cancer. J. Ovarian Res..

[15] De Decker K., Jaroch K.H., Bart J., Kooreman L.F.S., Kruitwagen R.F.P.M., Nijman H.W., Kruse A.J. (2021). Borderline ovarian tumor frozen section diagnoses with features suspicious of invasive cancer: A retrospective study. J. Ovarian Res..

[16] Tal O., Ganer Herman H., Gluck O., Levy T., Kerner R., Bar J., Sagiv R. (2020). Characteristics and prognosis of borderline ovarian tumors in pre and postmenopausal patients. Arch. Gynecol. Obstet..

[17] Shen F., Chen S., Gao Y., Dai X., Chen Q. (2017). The prevalence of malignant and borderline ovarian cancer in pre- and post-menopausal Chinese women. Oncotarget.

[18] Pecorino B., Laganà A.S., Mereu L., Ferrara M., Carrara G., Etrusco A., Di Donna M.C., Chiantera V., Cucinella G., Barra F. (2023). Evaluation of Borderline Ovarian Tumor Recurrence Rate after Surgery with or without Fertility-Sparing Approach: Results of a Retrospective Analysis. Healthcare.

[19] Baykuş Y., Deniz R., Çelik Kavak E. (2019). Factors affecting compliance of intraoperative frozen and final histopathology in borderline ovarian tumors: Retrospective cohort study. J. Surg. Med. Nisan.

[20] Sağır U., Kılıç Ç., Özkan H.D., Kılıç F., Ünsal M., Aytekin O., Mesci Ç., Cömert G.K., Turan T. (2022). Factors Predicting Inaccuracy Between Frozen Section Analysis and Postoperative Pathology Results: A Tertiary Center Experience. Hitit Med. J..

[21] Raimondo D., Raffone A., Scambia G., Maletta M., Lenzi J., Restaino S., Mascilini F., Trozzi R., Mauro J., Travaglino A. (2022). The impact of hysterectomy on oncological outcomes in postmenopausal patients with borderline ovarian tumors: A multicenter retrospective study. Front. Oncol..

[22] Huang Z., Li L., Li C., Ngaujah S., Yao S., Chu R., Xie L., Yang X., Zhang X., Liu P. (2018). Diagnostic accuracy of frozen section analysis of borderline ovarian tumors: A meta-analysis with emphasis on misdiagnosis factors. J. Cancer.

[23] Shao H., Wang N., Liu G. (2024). Factors Affecting the Diagnostic Discordance Between Frozen and Permanent Sections in Mucinous Ovarian Tumors. Int. J. Womens Health.

[24] Spaan M., van den Belt-Dusebout A.W., Lambalk C.B., van Boven H.H., Schats R., Kortman M., Broekmans F.J.M., Laven J.S.E., van Santbrink E.J.P., Braat D.D.M. (2021). Long-Term Risk of Ovarian Cancer and Borderline Tumors After Assisted Reproductive Technology. J. Natl. Cancer Inst..

[25] Akalin M., Akalin E.E., Giray B., Kocakusak C.K. (2021). Challenges of Frozen Section in Borderline Ovarian Tumors: A 10-Year Retrospective Analysis from a Tertiary Gynecological Cancer Center: Retrospective Analysis. J. Clin. Obstet. Gynecol..

[26] Zhang W., Jia S., Xiang Y., Yang J., Jia C., Leng J. (2019). Factors associated with misdiagnosis of frozen section of mucinous borderline ovarian tumor. J. Int. Med. Res..

[27] Chen Y.Y., Lin H., Wu C.H., Lan J., Tsai C.C., Fu H.C., Ou Y.C. (2021). Factors associated with the inconsistent diagnosis between frozen section and permanent pathologic examination in borderline ovarian tumors. J. Obstet. Gynaecol. Res..

[28] Yegin G.F., Taş E.E., Kılıç G., Keskin H.L., Yavuz A.F. (2020). Discriminating Borderline Ovarian Tumors from Ovarian Cancer: Focus on Systemic İnflammatory Response Markers. Cyprus J. Med. Sci..

[29] Song L., Wu Q., Bai S., Zhao J., Qi J., Zhang J. (2024). Comparison of the diagnostic efficacy of systemic inflammatory indicators in the early diagnosis of ovarian cancer. Front. Oncol..

[30] Barik A., Rashmi A., Singh V., Kumar Upadhyay A. (2024). C-Reactive Protein/Albumin Ratio and Clinicopathological Features in Ovarian Cancer: A Prospective Study. Cureus.

